# Complete mitochondrial genome of the longicorn *Anoplophora horsfieldi* Hope (Coleoptera: Cerambycidae)

**DOI:** 10.1080/23802359.2021.1915717

**Published:** 2023-02-02

**Authors:** Bo-Long Zhang, Jie Zhang, Dajiang Zhang, Ying Feng, Jing Qiu, Xiao-Jun Ye, Bao-Xin Wang

**Affiliations:** a Chengdu Academy of Agricultural and Forestry Sciences, Chengdu, China; b Jintang County Planning and Natural Resources Bureau, Chengdu, China

**Keywords:** *Anoplophora horsfieldi*, mitochondrial genome, phylogenetic analysis

## Abstract

The chinaberry yellow-banded longhorn beetle, *Anoplophora horsfieldi* Hope 1842 (Coleoptera: Cerambycidae) is an important pest on many economic tree species. In this study, the complete mitochondrial genome of *A. horsfieldi* was determined, which was 15,837 bp in length and contained 37 genes, including 13 protein-coding genes (PCGs), two rRNA, 22 tRNA genes, and a non-coding A + T-rich region. The phylogenetic analysis based on mitochondrial genomes showed that *A. horsfieldi* is sister to a clade formed by *A. chinensis* and *A. glabripennis*.

The chinaberry yellow-banded longhorn beetle, *Anoplophora horsfieldi* Hope 1842 (Coleoptera: Cerambycidae), is widely distributed in south China, Vietnam and India. It is an important pest on many economic tree species (Hua et al. 2009; Wang 2014). Here, we sequenced and annotated the complete mitochondrial genome of *A. horsfieldi*. The studied adult specimen was collected from Wenjiang District, Chengdu City, Sichuan Province, China (N30°41′47″, E103°50′45″) in 20 June 2020 and deposited at The Insect Specimen Room of Chengdu Academy of Agricultural and Forestry Sciences (http://www.cdnky.com/, Bo-Long Zhang and 978732857@qq.com) under the voucher no. D015016.1. Genomic DNA was extracted from *A. horsfieldi* legs using 2xCTAB method, and then sequenced using the Illumina NovaSeq platform. The Illumina sequencing generated 44 million of high-quality 150 bp paired-end reads, which were mapped to the published *A. chinensis* mitochondrial genome as the reference using mummer v3.1 (Kurtz et al. 2004). Then, A5-miseq v20150522 (Coil et al. 2014) and SPAdes v3.9.0 (Bankevich et al. 2012) were employed to assemble these reads into complete mitochondrial genome, of which the protein-coding genes (PCGs), transfer RNA genes (tRNAs), and ribosomal RNA genes (rRNAs) were predicted using the MITOZ software (https://github.com/linzhi2013/MitoZ) (Meng et al. 2019).

The circular mitochondrial genome of *A. horsfieldi* was 15,837 bp in length (GenBank accession number MW364565), and the nucleotide composition was significantly AT-biased (A: 39.65%, G: 12.44%, C: 8.16%, and T: 39.75%). The AT-skew and GC-skew of this genome were −0.001 and 0.207, respectively. The genome consisted of 37 genes, including 13 PCGs, two rRNAs, 22 tRNAs, and a non-coding A + T-rich region. Twenty-three genes were transcribed on the J-strand, whereas the others were oriented on the N-strand. The PCGs used four different start codons: ATT, ATG, ATA, or ATC, and two stop codons: TAA or TAG, except that *ND4* and *ND5* ended with TA, T completed by post transcriptional addition of 3′ A residues. The gene arrangement of *A. horsfieldi* mitochondrial genome was identical to that of other coleopterans. In order to validate the phylogenetic position of *A. horsfieldi*, the mitochondrial genomes of 13 representatives of the family Cerambycidae were used to construct a maximum-likelihood (ML) tree with *Aclees cribratus* (Curculionidae) as the outgroup. The nucleotide sequences of the 13 PCGs, aligned and concatenated in PhyloSuite v1.2.2 (Zhang et al. 2020) were used to perform the ML inference in IQ-TREE Multicore version 1.6.12 (Gao et al. 2018). The result indicated that *A. horsfieldi* is sister to a clade formed by *A. chinensis* and *A. glabripennis* (Figure 1).

**Figure 1.**
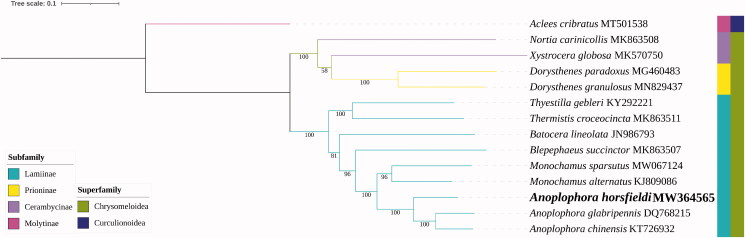
Maximum-likelihood phylogenetic analysis of 13 species of Cerambycidae and *Aclees cribratus* as outgroup based on the nucleotide sequences of the 13 PCGs from the mitochondrial genomes by IQ-TREE multicore version 1.6.12 under GTR + R6 model for 5000 ultrafast bootstraps and 5000 SH-aLRT test. Bootstrap values were shown next to nodes. GenBank accession numbers of each species were listed in the tree.

## Data Availability

The genome sequence data that support the findings of this study are openly available in GenBank of NCBI at https://www.ncbi.nlm.nih.gov/ under the accession no. MW364565. The associated BioProject, SRA, and Bio-Sample numbers are PRJNA693416, SRR13508141, and SAMN17386627, respectively.
